# Stereotactic Body Radiation Therapy vs. Transarterial Chemoembolization in Inoperable Barcelona Clinic Liver Cancer Stage a Hepatocellular Carcinoma: A Retrospective, Propensity-Matched Analysis

**DOI:** 10.3389/fonc.2020.00347

**Published:** 2020-03-24

**Authors:** Ting-Shi Su, Ping Liang, Ying Zhou, Yong Huang, Tao Cheng, Song Qu, Long Chen, Bang-De Xiang, Chang Zhao, De-Jia Huang, Shi-Xiong Liang, Le-Qun Li

**Affiliations:** ^1^Department of Radiation Oncology, Guangxi Medical University Cancer Hospital, Nanning, China; ^2^Department of Radiation Oncology, Rui Kang Hospital, Guangxi Traditional Chinese Medical University, Nanning, China; ^3^Department of Hepatobiliary Surgery, Guangxi Medical University Cancer Hospital, Nanning, China; ^4^Department of Interventional Radiology, Guangxi Medical University Cancer Hospital, Nanning, China

**Keywords:** hepatocellular carcinoma, SBRT, TACE, Barcelona Clinic Liver Cancer Stage A, overall survival

## Abstract

**Background and Objective:** It is unclear if stereotactic body radiation therapy (SBRT) or transarterial chemoembolization (TACE) is better for the treatment of inoperable early-stage hepatocellular carcinoma (HCC). This study aimed to retrospectively compare the efficacy of SBRT to TACE in patients with inoperable Barcelona Clinic Liver Cancer (BCLC)-A stage HCC.

**Materials and Methods:** In this multi-institutional retrospective study, a total of 326 patients with inoperable BCLC-A stage HCC were enrolled. Totally, 167 patients initially received SBRT and 159 initially received TACE. Overall survival (OS), local control (LC), intrahepatic control (IC), and progression-free survival (PFS) were evaluated in univariable and propensity-score matched analyses.

**Results:** There was a smaller median tumor size in the SBRT group than in the TACE group (3.4 cm vs. 7.2 cm, *P* < 0.001). After propensity score matching in the selection of 95 patient pairs, SBRT had better LC, IC, and PFS than TACE but showed comparable OS. The accumulative 1-, 3-, and 5-year OS rates were 85.7, 65.1, and 62.8% in the SBRT group and 83.6, 61.0, and 50.4% in the TACE group, respectively (*P* = 0.29). The accumulative 1-, 3-, and 5-year PFS were 63.4, 35.9, and 27.5% in the SBRT group and 53.5, 27.4, and 14.2% in the TACE group, respectively (*P* = 0.049). The accumulative 1-, 3-, and 5-year LC were 86.8, 62.5, and 56.9% in the SBRT group and 69.3, 53.3, and 36.6% in the TACE group, respectively (*P* = 0.0047). The accumulative 1-, 3-, and 5-year IC were 77.3, 45.9, and 42.4% in the SBRT group and 57.3, 34.1, and 17.7% in the TACE group, respectively (*P* = 0.003). On multivariate analysis, treatment (SBRT vs. TACE) was a significant covariate associated with local and intrahepatic control (HR = 1.59; 95% CI: 1.03–2.47; *P* = 0.04; HR = 1.61; 95% CI: 1.13–2.29; *P* = 0.009).

**Conclusions:** SBRT was an alternative to TACE for inoperable BCLC-A stage HCC with better local and intrahepatic control. Controlled clinical trials are recommended to evaluate the actual effects of this novel regimen adequately.

## Introduction

Patients with early-stage hepatocellular carcinoma (HCC) are candidates for potentially curative treatment options, such as liver transplantation, liver resection, and radiofrequency ablation, and they have a 5-year survival rate of 40–70% (1). However, some patients with Barcelona Clinic Liver Cancer (BCLC) stage A disease refuse to undergo surgery, or the procedure may be deemed too high risk for them. There is an urgent clinical need for a more effective therapy for HCC. Transarterial chemoembolization (TACE) is an established local treatment for patients with unresectable and non-transplantable stages of HCC with compensated liver disease and without extrahepatic spread (2, 3). Although the aforementioned conditions define BCLC stage B disease, TACE can also be applied to those with earlier-stage (BCLC stage A) disease who are not considered for surgery or ablation (4, 5).

Stereotactic body radiation therapy (SBRT) is an advanced external beam radiation therapy technique that delivers large ablative doses of radiation and low fractionation (6). There is increasing evidence (primarily from non-randomized controlled trials) supporting the clinical application of SBRT as a non-invasive treatment in patients with unresectable or recurrent HCC (7–10). SBRT can provide encouraging outcomes comparable to those associated with curative treatment options, including liver resection and radiofrequency ablation (11–13). Several studies have reported good clinical outcomes using SBRT in HCC with or without TACE (14–17). However, few comparative studies have analyzed the use of SBRT vs. TACE in BCLC-A stage HCC. In this retrospective study, we aimed to compare the long-term survival rates after SBRT and TACE in patients with early-stage HCC who were ineligible for resection or ablation therapies.

## Materials and Methods

### Patients

Datasets from January 2009 to January 2017 from two different institutions with a tertiary-A hospital in the Guangxi region of China, Cancer Hospital and Rui Kang Hospital, were used in this study. All cases of TACE were collected from Cancer Hospital, and cases of SBRT were collected from Rui Kang Hospital.

The eligibility criterion was the presence of BCLC stage A HCC in patients who were not considered for surgery, or refused to undergo surgery and/or local radiofrequency ablative therapies, and received SBRT or TACE as initial treatment. HCC diagnosis was established based on histopathology or according to the clinical criteria for HCC diagnosis (18). Exclusion criteria were as follows: (a) recurrence after other treatments, (b) intrahepatic cholangiocellular carcinoma, (c) gallbladder cancer, (d) liver metastases, and/or (e) prior history of conventional abdominal radiotherapy. All hospital charts and patients' documents were carefully reviewed.

### Transarterial Chemoembolization (TACE)

A French catheter (4 F−5 F) was inserted into the abdominal aorta through the right femoral artery using the Seldinger technique. Selective arteriography of the hepatic artery was carried out for tumor location. Hepatic angiography was performed for the detection of any obvious tumor staining in the remaining liver. Subsequently, an emulsion of oxaliplatin or lobaplatin or cisplatinum (20–100 mg), pharmorubicin or pirarubicin (10–40 mg), and lipiodol (2–15 ml) was infused via the catheter (15, 19). The effect of TACE was evaluated by computed tomography (CT) at the 1-month follow-up. Treatment was repeated one to six times (median, 3) at 3–6-week intervals in the TACE group.

### Stereotactic Body Radiation Therapy (SBRT)

SBRT was performed as described (7, 11, 20). Briefly, gross tumor volume was outlined under the fusion image of CT and magnetic resonance imaging (MRI) by comparing different imaging phases, and the gross tumor volume was expanded by 0–5 mm for the formation of the planning target volume (PTV). SBRT was implemented using a CyberKnife system (Accuray Inc., Sunnyvale, CA, USA). A 28–50 Gy dose of radiation was delivered in one to five fractions on consecutive days at the 55–80% isodose line that covered at least 97% of the planning target volume. Fractionation schedules and total doses were chosen according to the tumor size and dose–volume constraints of the organs at risk.

### Response Evaluation and Follow-Up

The evaluations included laboratory tests and imaging with contrast-enhanced CT and/or MRI at 1 month after the procedure and every 3–6 months thereafter. The laboratory examinations assessed levels of alanine transaminase (ALT), aspartate transaminase (AST), prothrombin time, levels of total bilirubin, albumin, and alpha-fetoprotein. The modified Response Evaluation Criteria in Solid Tumors guideline was used to describe changes in the treated areas (21). Local recurrence/progress was defined as the reappearance of radiologic hallmarks of HCC for in-field-treated PTV lesions and/or progressive increase in tumor sizes during follow-up. Intrahepatic recurrence was defined as the reappearance of radiologic hallmarks of HCC (hypervascularity in the arterial phase with washout in the portal venous or delayed phases) and out-field-treated (PTV) lesions in the whole parenchyma of the liver. For progressive increase in tumor sizes without typical CT/MRI characteristics, diagnosis was confirmed by histopathology.

### Statistical Analysis

R version 3.6.1 software (2019 Microsoft Corporation) was used for the statistical analysis. *P* < 0.05 was considered statistically significant. Kaplan–Meier curves with the log-rank test were used to calculate patients' overall survival (OS), local control (LC) rate, intrahepatic control (IC) rate, and progression-free survival (PFS). In addition, accumulative overall survival (OS) was calculated starting from the date of the first treatment to death from any cause, with patients censored at the end of the study (April 11, 2019). Accumulative PFS was calculated starting from the date of the first treatment to the date of any tumor recurrence, progression, or death or the date of censoring. Accumulative LC was calculated starting from the date of the first treatment to the date of local tumor failure or the date of censoring. Accumulative IC was calculated starting from the date of the first treatment to the date of intrahepatic tumor failure or the date of censoring.

Variables without associations between each other by chi-squared/Mann–Whitney tests (Figure S1). We use univariate and least absolute shrinkage and selection operator (LASSO) to identify non-associated predictive variables that contribute toward the final multivariate. The report concordance index gives an indication of the predictive fit (Figure S2).

To reduce selection bias and potential confounding effect of treatment, a 1:1 nearest neighbor matching that pairs patients who have the closest propensity scores was performed to create a balanced cohort. The logit of the propensity score for matching was used with a caliper of 0.2 times its standard deviation as recommended by Austin (22), based on the potential confounding variables including age, gender, ALBI score, ALT, tumor size, and alpha-fetoprotein (AFP) levels. Only patients who were matched were included (Figure S3).

## Results

### Patients

During the study period from January 2009 to January 2017, 326 patients with BCLC stage A HCC, who were not considered for surgery and/or radiofrequency ablative therapies were enrolled retrospectively. A total of 167 patients initially received SBRT, and 159 initially received TACE. Some variables differed between the groups, including age, ALT levels, tumor size, ALBI score, and AFP level. After propensity score matching, 95 paired patients were selected from the SBRT and TACE groups. There was no significant difference between the groups, and the balance of variables in the matched cohorts was markedly improved (Table 1).

**Table 1:** Patient and treatment characteristics for different treatment groups.

		**Before propensity matching**			**After propensity matching**			
**Factor**	**Level**	**SBRT**	**TACE**	***p*-value**	**SBRT**	**TACE**	***p*-value**	**Test**
Number of patients		167	159		95	95		
Gender	Male	141 (84.4%)	139 (87.4%)	0.44	83 (87%)	84 (88%)	0.82	Pearson's chi-squared
Age, median (IQR)		56 (47, 65)	52 (44, 61)	0.007	55 (45, 63)	52 (44, 63)	0.31	Wilcoxon rank-sum
Age	>/=60	70 (41.9%)	49 (30.8%)	0.037	36 (38%)	29 (31%)	0.28	Pearson's chi-squared
HBV	Positive	145 (86.8%)	141 (88.7%)	0.61	83 (87%)	84 (88%)	0.82	Pearso's chi-squared
	Negative	22 (13.2%)	18 (11.3%)		12 (13%)	11 (12%)		
Tbil, median (IQR)		13.3 (9.3, 20.1)	14.4 (9.3, 21.5)	0.48	13.4 (9.7, 20.5)	14.9 (9.3, 22.4)	0.52	Wilcoxon rank-sum
Albumin, median (IQR)		37.9 (34.5, 41.7)	38.7 (34.2, 42)	0.63	37.6 (34.4, 41.8)	39.3 (33.9, 42.3)	0.26	Wilcoxon rank-sum
ALT, median (IQR)		31 (21, 44)	44 (35, 82)	<0.001	35 (23, 50)	39 (32, 60)	0.1	Wilcoxon rank-sum
AST, median (IQR)		31 (21, 50)	35 (24, 53)	0.13	36 (23, 52)	31 (22, 43)	0.24	Wilcoxon rank-sum
PT, median (IQR)		13.2 (12.5, 14)	13.1 (12.3, 14.4)	0.97	13.1 (12.5, 14.1)	13.3 (12.4, 14.8)	0.41	Wilcoxon rank-sum
Size, median (IQR)		3.4 (2.4, 5.2)	7.2 (4.2, 12.1)	<0.001	4.5 (3, 6.7)	5 (3, 7.1)	0.6	Wilcoxon rank-sum
Size status	1–5 cm	123 (73.7%)	53 (33.3%)	<0.001	53 (56%)	49 (52%)	0.47	Pearson's chi-squared
	5–10 cm	40 (24.0%)	54 (34.0%)		38 (40%)	38 (40%)		
	10–19.5 cm	4 (2.4%)	52 (32.7%)		4 (4%)	8 (8%)		
ALBI score, median (IQR)		−2.519 (−2.802, −2.179)	−2.511 (−2.878, −2.127)	0.88	−2.515 (−2.761, −2.127)	−2.530 (−2.967, −2.088)	0.5	Wilcoxon rank-sum
ALBI grade	1	73 (43.7%)	64 (40.3%)	0.48	41 (43%)	40 (42%)	0.29	Pearson's chi-squared
	2	88 (52.7%)	85 (53.5%)		51 (54%)	47 (49%)		
	3	6 (3.6%)	10 (6.3%)		3 (3%)	8 (8%)		
Child–Pugh score	5	105 (62.9%)	118 (74.2%)	0.2	59 (62%)	70 (74%)	0.24	Pearson's chi-squared
	6	32 (19.2%)	21 (13.2%)		17 (18%)	11 (12%)		
	7	18 (10.8%)	9 (5.7%)		12 (13%)	5 (5%)		
	8	5 (3.0%)	4 (2.5%)		3 (3%)	3 (3%)		
	9	7 (4.2%)	7 (4.4%)		4 (4%)	6 (6%)		
Child–Pugh class	A	137 (82.0%)	139 (87.4%)	0.18	76 (80%)	81 (85%)	0.34	Pearson's chi-squared
	B	30 (18.0%)	20 (12.6%)		19 (20%)	14 (15%)		
AFP	0–7	58 (34.7%)	35 (22.0%)	0.013	28 (29%)	22 (23%)	0.61	Pearson's chi-squared
	>7–100	50 (29.9%)	42 (26.4%)		23 (24%)	28 (29%)		
	>100–400	20 (12.0%)	22 (13.8%)		15 (16%)	12 (13%)		
	>400	39 (23.4%)	60 (37.7%)		29 (31%)	33 (35%)		

*ALBI, albumin–bilirubin; AFP, alpha fetoprotein; TACE, transarterial embolization; SBRT, stereotactic body radiation therapy; PT, prothrombin time; AST, aspartate aminotransferase; ALT, alanine aminotransferase*.

### SBRT vs. TACE

This study was concluded on April 11, 2019. The median follow-up time was 35.0 months in the SBRT group and 32.0 months in the TACE group. A total of 50 cases died, and 117 cases were right-censored, including 31 cases lost to follow-up, while 86 cases were still alive at the end of the study in the SBRT group. On the other hand, 66 cases died, and 96 cases were right-censored, including 41 cases lost to follow-up, while 55 cases were still alive at the end of the study in the TACE group. During the follow-up period, the total local and intrahepatic recurrence after SBRT was lower than that after TACE (69/167 vs. 98/159, *P* = 0.037).

**Figure 1:**
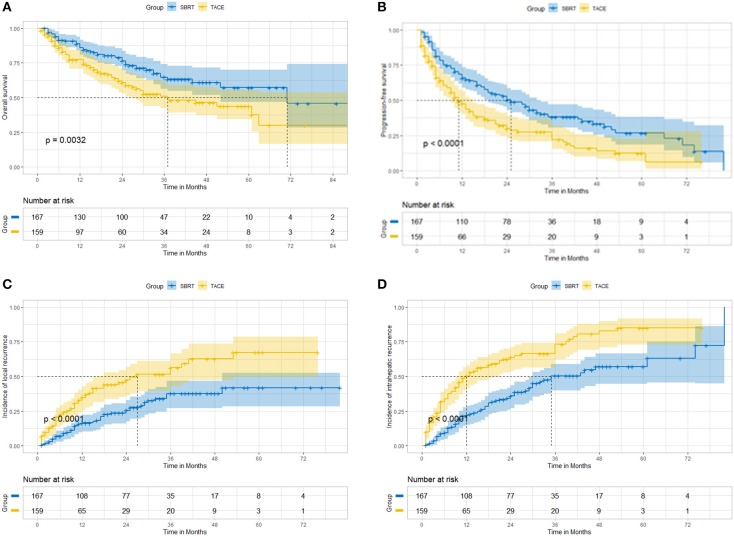
Before propensity matching, SBRT vs. TACE. **(A)** overall survival; **(B)** progression-free survival; **(C)** local control; **(D)** intrahepatic control.

Before propensity score matching, the accumulative OS (Figure 1A), PFS (Figure 1B), LC (Figure 1C), and IC (Figure 1D) at 12, 36, and 60 months were better in patients undergoing SBRT than in those undergoing TACE (Table 2). The accumulative 3-year OS was 64.7% in the SBRT group and 51.0% in the TACE group (*P* = 0.005, HR = 1.71, 95% CI: 1.17–2.51). The accumulative 3-year PFS was 38.1% in the SBRT group and 27.6% in the TACE group (*P* = 0.0005, HR = 1.62, 95% CI: 1.22–2.16). The accumulative 3-year LC was 63.1% in the SBRT group and 50.2% in the TACE group (*P* = 0.0008, HR = 1.93, 95% CI: 1.29–2.88). The accumulative 3-year IC 49.6% in the SBRT group and 33.5% in the TACE group (*P* < 0.0001, HR = 2.14, 95% CI: 1.54–2.96).

**Table 2:** The accumulative OS, PFS, and local and intrahepatic control of different treatment groups.

		**Before propensity matching**					**After propensity matching**				
	**Months**	**SBRT**	**TACE**	**HR**	**95% CI**	***P***	**SBRT**	**TACE**	**HR**	**95% CI**	***P***
OS	12	86.0%	77.3%	1.79	1.05–3.03	0.031	85.7%	83.6%	1.19	0.57–2.49	0.65
	36	64.7%	51.0%	1.71	1.17–2.51	0.005	65.1%	61.0%	1.17	0.7–1.96	0.55
	60	57.3%	43.8%	1.69	1.17–2.46	0.0046	62.8%	50.4%	1.19	0.79–2.14	0.3
PFS	12	65.9%	47.5%	1.82	1.30–2.57	0.0003	63.4%	53.3%	1.46	0.93–2.28	0.092
	36	38.1%	27.6%	1.62	1.22–2.16	0.0005	35.9%	27.4%	1.37	0.95–1.97	0.081
	60	26.7%	12.4%	1.71	1.30–2.25	0.0001	27.5%	14.2%	1.44	1.01–2.04	0.037
LC	12	85.1%	67.2%	1.79	1.05–3.03	0.0031	86.8%	69.3%	1.93	1.18–3.18	0.0035
	36	63.1%	50.2%	1.93	1.29–2.88	0.0008	62.5%	53.5%	1.81	1.08–3.04	0.0219
	60	59.3%	35.6%	2.04	1.38–3.01	0.0002	56.9%	36.6%	1.93	1.18–3.18	0.0084
IC	12	77.8%	49.6%	2.94	1.99–4.32	0.0001	77.3%	57.3%	2.33	1.39–3.93	0.0016
	36	49.6%	33.5%	2.14	1.54–2.96	0.0001	45.9%	34.1%	1.7	1.12–2.58	0.01
	60	42.9%	15.1%	2.32	1.69–3.17	0.0001	42.4%	17.7%	1.85	1.23–2.77	0.0021

**Figure 2:**
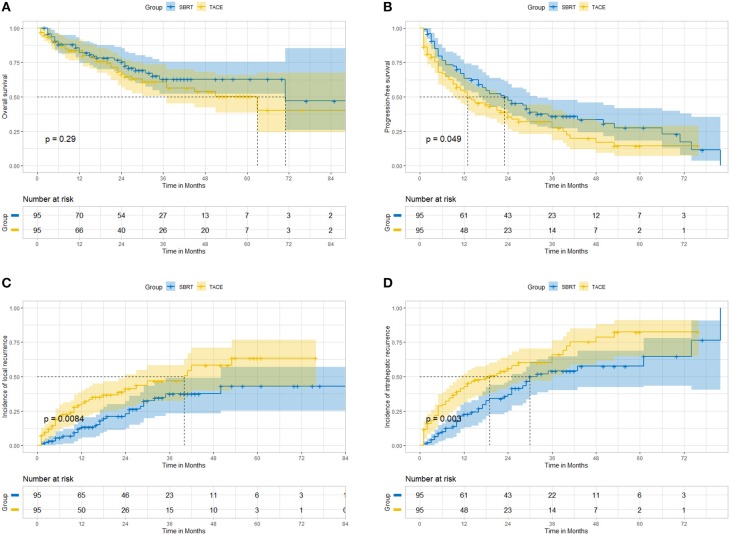
After propensity matching, SBRT vs. TACE. **(A)** overall survival; **(B)** progression-free survival; **(C)** local control; **(D)** intrahepatic control.

After propensity score matching in the selection of 95 well-pairs patients, we found no statistically significant difference in OS (Figure 2A); patients in the SBRT group had better long-term PFS (Figure 2B), LC (Figure 2C), and IC (Figure 2D) than those in the TACE group (Table 2). The accumulative 3-year OS was 65.1% in the SBRT group and 61.0% in the TACE group (*P* = 0.55, HR = 1.17, 95% CI: 0.7–1.96). The accumulative 3-year PFS was 35.9% in the SBRT group and 27.4%, in the TACE group (*P* = 0.081, HR = 1.37, 95% CI: 0.95–1.97). The accumulative 3-year LC was 62.5% in the SBRT group and 53.5% in the TACE group (*P* = 0.0219, HR = 1.81, 95% CI: 1.08–3.04). The accumulative 3-year IC was 45.9% in the SBRT group and 34.1% in the TACE group (*P* = 0.01, HR = 1.70, 95% CI: 1.12–2.58).

### Multivariable Cox Analysis

**Figure 3:**
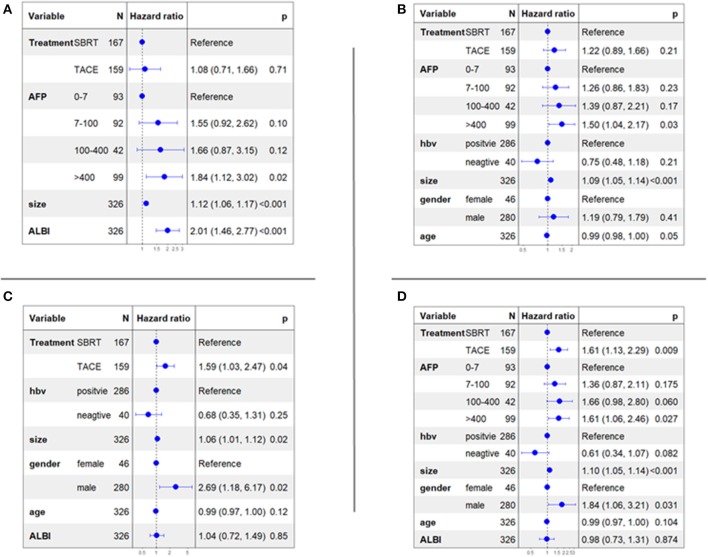
Multivariable cox analyses of all patients. **(A)** overall survival; **(B)** progression-free survival; **(C)** local control; **(D)** intrahepatic control.

Cox proportional hazards models accounting for clustering were used to compare the two treatment groups. The selection of influencing factors, which were considered for multivariate analysis, was based on LASSO analysis (Table 3). Multivariable cox regression analysis of OS (Figure 3A) showed that three independent predictors were size (HR = 1.12; 95% CI, 1.06–1.17; *P* < 0.001), ALBI score (HR = 2.01; 95% CI: 1.46–2.77; *P* < 0.001), and AFP level >400 (HR = 1.84; 95% CI: 1.12–3.02; *P* = 0.02). Multivariable cox regression analysis of PFS (Figure 3B) showed that three independent predictors were size (HR = 1.09; 95% CI: 1.05–1.14; *P* < 0.001), age (HR = 0.99; 95% CI: 0.98–1.00; *P* = 0.05), and AFP level >400 (HR = 1.52; 95% CI: 1.50–2.17; *P* = 0.03). Multivariable cox regression analysis of LC (Figure 3C) showed that three independent predictors were size (HR = 1.06; 95% CI: 1.01–1.12; *P* = 0.02), gender/male (HR = 2.69; 95% CI: 1.18–6.17; *P* = 0.02), and treatment (SBRT vs. TACE) (HR = 1.59; 95% CI: 1.03–2.47; *P* = 0.04). Multivariable cox regression analysis of IC (Figure 3D) showed that four independent predictors were size (HR = 1.10; 95% CI: 1.05–1.14; *P* < 0.001), gender/male (HR = 1.84; 95% CI: 1.06–3.21; *P* = 0.031), AFP level >400 (HR = 1.61; 95% CI: 1.06–2.46; *P* = 0.027), and treatment (SBRT vs. TACE) (HR = 1.61; 95% CI: 1.13–2.29; *P* = 0.009).

**Table 3:** Univariable and multivariable Cox analyses for OS, PFS, and local and intrahepatic control.

				**OS**									**PFS**							
				**Univariable analysis**				**Multivariable analysis**					**Univariable analysis**				**Multivariable analysis**			
**Factor**	**Level**	***N***	***n***	**HR**	***P***	**95% CI**		**HR**	***P***	**95% CI**		***n***	**HR**	**P**	**95% CI**		**HR**	***P***	**95% CI**	
Age		326	116	0.992	0.297	0.977	1.007					216	0.980	0.001	0.969	0.991	0.990	0.050	0.975	0.999
Gender	Female	46	17	1.000								27	1.000				1.000			
	Male	280	99	1.005	0.986	0.600	1.682					189	1.331	0.165	0.889	1.994	1.190	0.410	0.790	1.790
HBV	Positive	268	102	1.000								194	1.000				1.000			
	Negative	58	14	0.905	0.726	0.517	1.583					22	0.720	0.146	0.463	1.120	0.750	0.210	0.480	1.180
AFP	0–7	93	26	1.000				1.000				57	1.000				1.000			
	>7–100	92	33	1.446	0.160	0.864	2.421	1.550	0.100	0.920	2.620	59	1.146	0.467	0.794	1.653	1.260	0.230	1.260	0.230
	>100–400	42	15	1.477	0.229	0.782	2.791	1.660	0.120	0.870	3.150	27	1.338	0.216	0.844	2.121	1.390	0.170	0.870	2.210
	>400	99	42	2.041	0.004	1.249	3.333	1.840	0.020	1.120	3.020	73	1.796	0.001	1.264	2.552	1.502	0.030	1.040	2.170
PT		326	116	1.165	0.001	1.061	1.279					216	1.036	0.402	0.954	1.125				
Tbil		326	116	1.003	0.419	0.996	1.010					216	0.998	0.663	0.991	1.006				
Albumin		326	116	0.937	0.000	0.908	0.966					216	0.985	0.204	0.962	1.008				
AST		326	116	1.003	0.170	0.999	1.007					216	1.003	0.053	1.000	1.006				
ALT		326	116	1.003	0.034	1.000	1.007					216	1.003	0.030	1.000	1.005	1.000	0.929	0.997	1.003
Child-Pugh socre		326	116	1.294	0.001	1.116	1.499					216	1.098	0.142	0.969	1.243				
ALBI score		326	116	1.933	0.000	1.430	2.613	2.010	0.000	1.460	2.770	216	1.159	0.229	0.911	1.476				
Tumor size		326	116	1.120	0.000	1.073	1.168	1.120	0.000	1.060	1.170	216	1.107	0.000	1.072	1.143	1.090	0.000	1.046	1.140
Treatment	SBRT	167	50	1.000				1.000				104	1.000				1.000			
	TACE	159	66	1.723	0.004	1.192	2.490	1.080	0.710	0.710	1.660	112	1.723	0.000	1.314	2.258	1.220	0.210	0.890	1.660
				**LC**									**HC**							
				**Univariable analysis**				**Multivariable analysis**					**Univariable analysis**				**Multivariable analysis**			
**Factor**	**Level**	***N***	***n***	**HR**	***P***	**95% CI**		**HR**	***P***	**95% CI**		***n***	**HR**	***P***	**95% CI**		**HR**	***P***	**95% CI**	
Age		326	105	0.980	0.014	0.964	0.996	0.990	0.120	0.970	1.000	167	0.977	0.000	0.965	0.990	0.990	0.104	0.970	1.000
Gender	Female	46	6	1.000				1.000				14	1.000				1.000			
	Male	280	99	3.112	0.007	1.364	7.100	2.690	0.020	1.180	6.170	153	2.102	0.008	1.215	3.636	1.840	0.031	1.060	3.210
HBV	Positive	268	95	1.000				1.000				153	1.000				0.610	0.082	0.240	1.070
	Negative	58	10	0.690	0.265	0.360	1.325	0.680	0.250	0.350	1.310	14	0.595	0.063	0.344	1.029				
AFP	0–7	93	30	1.000								42	1.000				1.000			
	>7–100	92	29	1.106	0.698	0.663	1.846					44	1.237	0.328	0.808	1.896	1.360	0.175	0.870	2.110
	>100–400	42	11	0.921	0.816	0.462	1.839					23	1.598	0.073	0.957	2.668	1.660	0.060	1.980	2.800
	>400	99	35	1.542	0.084	0.944	2.517					58	1.980	0.001	1.322	2.964	1.610	0.027	1.060	2.460
PT		326	105	0.993	0.916	0.878	1.124					167	0.987	0.796	0.894	1.090				
Tbil		326	105	0.997	0.616	0.985	1.009					167	0.996	0.415	0.986	1.006				
Albumin		326	105	1.000	0.978	0.967	1.035					167	1.004	0.777	0.977	1.032				
AST		326	105	1.004	0.027	1.000	1.007					167	1.003	0.061	1.000	1.006				
ALT		326	105	1.003	0.057	1.000	1.006	1.001	0.698	0.997	1.005	167	1.004	0.004	1.001	1.006	1.001	0.644	0.998	1.004
Child–Pugh socre		326	105	1.036	0.719	0.856	1.253					167	0.948	0.520	0.804	1.117				
ALBI score		326	105	0.989	0.951	0.694	1.410	1.040	0.120	0.720	1.490	167	0.950	0.723	0.715	1.262	0.980	0.874	0.730	1.310
Tumor size		326	105	1.097	0.000	1.049	1.148	1.061	0.020	1.001	1.120	167	1.125	0.000	1.086	1.165	1.100	0.000	1.050	1.140
Treatment	SBRT	167	44	1.000				1.000				69	1.000				1.000			
	TACE	159	61	2.057	0.000	1.393	3.038	1.590	0.041	1.013	2.470	98	2.300	0.000	1.684	3.143	1.610	0.009	1.130	2.290

*ALBI, albumin–bilirubin; AFP, alpha fetoprotein; TACE, transarterial chemoembolization; SBRT, stereotactic body radiation therapy; PT, prothrombin time; AST, aspartate aminotransferase; ALT, alanine aminotransferase; HBV, hepatitis b virus; n, number of events; N, number in each category*.

In our matched cohort, multivariable cox regression analysis showed that ALBI score was the only independent predictor of OS (HR = 1.84; 95% CI: 1.23–2.76; *P* = 0.003). Tumor size was the only independent predictor of PFS (HR = 1.12; 95% CI: 1.06–1.19; *P* < 0.001). Treatment (SBRT vs. TACE) was the only independent predictor of LC (HR = 2.19; 95% CI: 1.27–3.77; *P* = 0.005). Treatment (SBRT vs. TACE) and tumor size were two independent predictors of IC (HR = 1.99; 95% CI: 1.30–3.04; *P* = 0.001; and HR = 1.09; 95% CI: 1.02–1.16; *P* = 0.014).

In the SBRT cohort, multivariable cox regression analysis showed that AFP level and ALBI score were two independent predictors of OS (HR = 1.34; 95% CI: 1.04–1.73; *P* = 0.02; and HR = 2.84; 95% CI: 1.72–4.71; *P* < 0.001). The included influencing factors were not prognostic factors for PFS, LC, and IC.

In the TACE cohort, multivariable cox regression analysis showed that tumor size was an independent predictor of OS (HR = 1.09; 95% CI: 1.04–1.13; *P* < 0.001). Tumor size and age were two independent predictors of PFS (HR = 1.84; 95% CI: 1.23–2.76; *P* = 0.003; and HR = 0.97; 95% CI: 0.95–0.99; *P* < 0.001). Age was an independent predictor of LC (HR = 0.97; 95% CI: 0.95–0.99; *P* = 0.02). Tumor size and age were two independent predictors of IC (HR = 1.10; 95% CI: 1.05–1.15; *P* < 0.001; and HR = 0.98; 95% CI: 0.96–0.99; *P* = 0.04).

### Complications and Mortality

Both treatment regimens have their own specific complications. In the TACE group, TACE-related deaths occurred in 2 (1.3%) of 159 patients after the initial TACE. The cause of death consisted of hepatic failure in one patient and liver abscess in the other. In the SBRT group, treatment-related deaths occurred in 2 (1.2%) of 167 patients after SBRT due to hepatic failure. Complications were graded according to the National Cancer Institute Common Terminology Criteria for Adverse Events version 4.03. The most common grades of acute complications in all groups were ≤3. Fever, hepatic pain, and increased levels of ALT or AST (≥3-fold) were the three most common TACE-related complications. Fatigue, nausea, and vomiting were the three most common SBRT-related complications. Elevated Child–Pugh score was common TACE or SBRT-related hepatotoxicity (+1 score: 28/159 vs. 20/167, *P* = 0.22; and +2 score: 8/169 vs. 17/167, *P* = 0.11). Most of the complications and hepatotoxicity were reversed by conservative and supportive treatment.

## Discussion

SBRT was shown to be an alternative option for patients with inoperable BCLC stage A disease. Our propensity match-based analysis after locoregional therapy for 326 inoperable patients with BCLC stage A disease in China, where hepatitis B virus (>86.8%) was predominant, suggests that patients undergoing SBRT have a similar OS to those undergoing TACE, with excellent local and intrahepatic control and PFS. On multivariate analysis, treatment (SBRT vs. TACE, HR = 1.59; 95% CI: 1.03–2.47; *P* = 0.04; HR = 1.61; 95% CI: 1.13–2.29; *P* = 0.009) and tumor size (HR = 1.06; 95% CI: 1.01–1.12; *P* = 0.02; HR = 1.10; 95% CI: 1.05–1.14; *P* < 0.001) were significant covariates associated with local and intrahepatic control. SBRT does not seem to compromise the measured survival outcomes after TACE. Sapir et al. (23) also reported that 209 patients with 287 HCC tumors, and 28 of these cases with portal vein branch thrombosis (BCLC-C), were treated with TACE (*n* = 84) or SBRT (*n* = 125) in western countries, where hepatitis C virus (70%) and alcohol abuse (20%) were the main cause of HCC. It was also found that SBRT can be an alternative to TACE for local HCC with one to two tumors and provided better LC, with no difference in OS. The 1- and 2-year OS were 74.1 and 34.9% after SBRT, and 75.3 and 54.9% after TACE, respectively. The 1- and 2-year LC were 47.1 and 22.9% after TACE compared to 96.5 and 91.3% after SBRT, respectively. The 1- and 2-year intrahepatic controls were better for patients after SBRT than after TACE (56.5, 26.9% vs. 35.9, 10.7%, respectively), favoring SBRT significantly (HR = 3.55, 95% CI 1.94–6.52, *P* < 0.001). In addition, higher AFP, previous treatment status, and branch thrombosis were significant covariates associated with intrahepatic control in multivariate analysis. Shen et al. compared the local control and overall survival between SBRT (*n* = 46) and TACE (*n* = 142) in medium-sized (3–8 cm) HCC in Taiwan. The 3-year local control rate was 73.3% for the SBRT group and 63.0% for the TACE group. Multivariable analyses also identified the independent predictors for local control as treatment modality (SBRT or TACE), gender (female vs. male), and recurrence HCC status (recurrence or primary diagnosis). After propensity score matching analysis, patients in the SBRT group also had better local control (3-year of 77.5 vs. 55.6%; *P* = 0.007) and OS (3-year OS of 55.0 vs. 13.0%; *P* < 0.001) than those in the TACE group. However, there was no difference in local control and OS between SBRT and TACE in newly diagnosed HCC cases. Sapisochin et al., in a retrospective study, reported that SBRT, RFA, and TACE were evaluated to have similar effectiveness in bridging therapy for liver transplant with tumor necrosis at explant, and overall survival (24).

According to BCLC guidelines, liver transplantation, liver resection, and RFA were recommended as first-line potentially curative treatment options for patients with early-stage HCC, providing a long-term survival at 5 years of more than 40–70% (1). TACE is reserved for patients with intermediate-stage multinodular HCC, Child–Pugh class A or B disease, and good performance status. It can also be applied to those with BCLC stage A disease who are not considered for surgery or ablation (4, 5). Burrel et al. (4) found that the 1-, 3-, and 5-year survival for such patients (*n* = 41) treated with drug-eluting beads (DEB-TACE) was 89.7, 67.8, and 33.9%, respectively. Takayasu et al. (5) found that the 1-, 3-, 5-, and 7-year survival rates for TNM stage I patients (*n* = 489) treated with TACE were 98, 78, 52, and 38%, respectively. Takaki et al. reported that 1-, 3-, 5-, and 10-year survival rates were 93.3%, 83.2, 61.5, and 17.6% in the T1 group, and 93.5, 68.7, 43.5, and 12.2% in the T2 group, respectively. The 2-, 3-, and 5- year local recurrence rates were 46, 58, and 63% in the whole group (25). In the current study, the accumulative 1-, 3-, and 5-year OS rates were 83.6, 61.0, and 50.4%, local recurrence rates were 30.7, 46.7, 63.4%, and intrahepatic recurrence rates were 42.7, 65.9, and 82.3%, respectively, in the TACE group after propensity score matching. Some retrospective studies showed that SBRT for primary HCC provides high rates of durable local control (80–100%) (7, 12, 26–28). Our LC rate in the SBRT group compared favorably to the published literature. Thus, given the agreement with previous literature, the higher rate of local and intrahepatic control after SBRT in this study is most likely due to a true difference in the treatment effectiveness, rather than an artifact from a particularly excellent SBRT or poor TACE procedures.

Tumor diameter was an independent prognostic factor of OS, PFS, and intrahepatic control in TACE group based on univariable and multivariable cox analyses. Lo et al. also reported that a tumor diameter of ≤5 cm was a good prognostic factor of TACE (7). Tumor control rates after TACE have varied considerably, even in prospective studies (2, 3, 29). In the use of interventional therapy to larger HCC, certain bottlenecks may be encountered. Embolization of the hepatic arteries could cause tumor necrosis because these arteries may feed nutrients to the tumor cells. However, the liver surrounding HCCs has arterial vessels and veins and, therefore, may not undergo complete necrosis due to arterial embolization alone. The long-term locoregional curative effect is unsatisfactory in cases with TACE alone, especially with large tumors, as tumor cells relapse from the intracapsular or extracapsular HCC invasion and can rarely be eradicated (30). TACE was feasible and associated with a higher response rate than that of TAE alone (31). DEB-TACE showed a better local response, lower recurrence rates, and longer time to progression than TACE (31). DEB-TACE was associated with a significant reduction in the occurrence of serious liver toxicity (32). However, no apparent differences in OS were observed between both treatment groups. These results challenge the use of DEB-TACE in HCC (33). Y90 radioembolization led to improved time to progression compared to TACE but did not improve OS (34).

Unlike the mechanism by which TACE works, radiotherapy has the advantage of directly damaging the tumor cells. On subgroup analysis, tumor diameter has a great influence on TACE survival time and intrahepatic control, while tumor diameter has little effect on SBRT. SBRT can also provide encouraging outcomes comparable to radiofrequency ablation (12, 35, 36). In our previous study, SBRT can provide encouraging curative outcomes comparable to liver resection for small HCC. The 5-year OS rates were 70.0 and 64.4% in the SBRT and liver resection groups, respectively (11). In the current study, we found that SBRT was superior to TACE, providing better local and intrahepatic control. In recent years, there have been several reports on the combination of TACE and SBRT, suggesting that the combination may yield better outcomes than TACE alone. Jun et al. reported that SBRT-TACE is superior to TACE regarding LC in patients with one or two small HCC lesions (37). Kimura et al. found that TACE+SBRT was not better than SBRT alone in small HCC cases (38). It may be concluded that SBRT has a radical curative effect in these selected HCCs. Additional multi-institutional prospective studies are warranted for the investigation of the real effects of SBRT. The TASABR randomized controlled trial is underway and will compare SBRT vs. re-TACE for HCC patients who had an incomplete response after initial TACE (39). A randomized phase 2 trial (NCT02182687), designed to compare TACE or SBRT as a bridge to transplant with the primary outcome as time to first additional intervention, is underway. Another phase 2 trial (NCT02470533), which targets patients with one to three tumors and assesses time to any progression after SBRT or after DEB-TACE, is ongoing. Our findings also need to be verified prospectively in HCC patients with BCLC-A stage who are not considered for surgery or have refused to undergo surgery and/or local radiofrequency ablative therapies.

This study has some limitations. First, this was a retrospective, non-randomized study, and the follow-up period was not lengthy, and this could have obscured late effects. Second, it is difficult to eliminate selection bias. Some variables differed between the groups; therefore, propensity score matching was applied at a 1:1 ratio to reduce selection bias and potential confounding effects of treatment. We also used the albumin–bilirubin score instead of the Child–Pugh score to reduce subjective errors. However, the bias of selection factors cannot be completely avoided. Controlled clinical trials are recommended to evaluate the actual effects of this novel regimen adequately. Third, we cannot account for differences between the two groups that are not known, such as the experience of institutions, patient's financial condition, and benefit of second-line treatment after recurrence.

In conclusion, SBRT was an alternative to TACE for inoperable BCLC-A stage HCC with excellent local and intrahepatic control. Controlled clinical trials are recommended to evaluate the actual effects of this novel regimen adequately.

## Data Availability Statement

The datasets analyzed in this study can be found with the corresponding author, Ting-Shi Su The statistical code and dataset are available from the author at sutingshi@163.com.

## Ethics Statement

This study complied with the tenets of the Declaration of Helsinki. The study design was approved by the ethics review board of Guangxi Medical University Cancer Hospital (LW2019036). The ethics committee waived the requirement of written informed consent for participation.

## Author Contributions

T-SS, S-XL, and L-QL made substantial contributions to the study's conception and design. T-SS, PL, YZ, YH, TC, CZ, D-JH, SQ, LC, and B-DX made substantial contributions to the acquisition of data. T-SS made substantial contributions to the data analysis and interpretation. All authors participated in the drafting of the article or revising it critically for important intellectual content, and they provided final approval of the version to be published.

### Conflict of Interest

The authors declare that the research was conducted in the absence of any commercial or financial relationships that could be construed as a potential conflict of interest.
