# Supplementary material for: Stereotactic Body Radiation Therapy vs. Transarterial Chemoembolization in Inoperable Barcelona Clinic Liver Cancer Stage a Hepatocellular Carcinoma: A Retrospective, Propensity-Matched Analysis
Source: Front Oncol. 2020 Mar 24;10:347. doi: 10.3389/fonc.2020.00347 (PMC7105822; doi:10.3389/fonc.2020.00347)

Fig S1: Report associations between variables

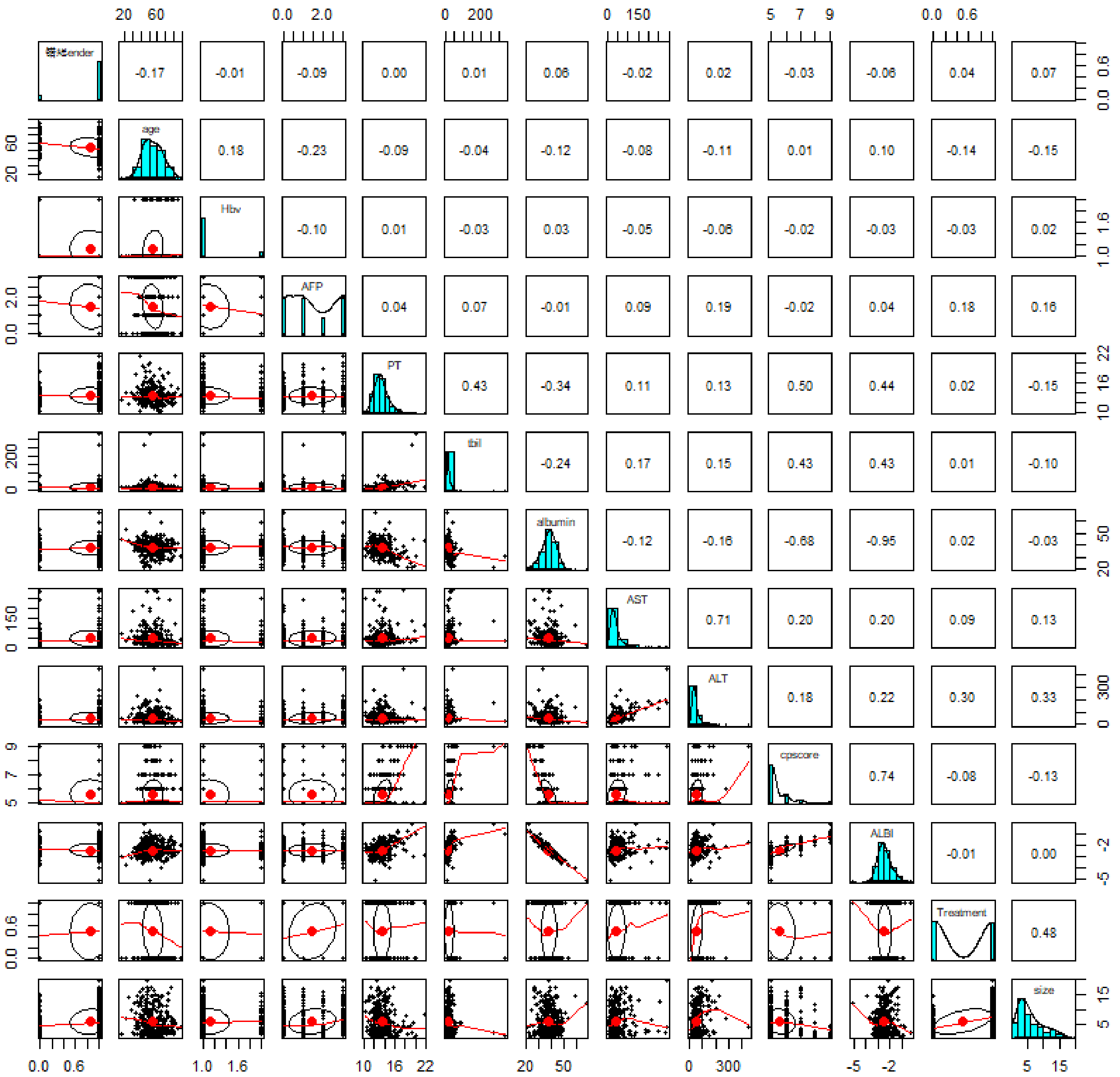

Fig S2: The report concordance index in 36 months

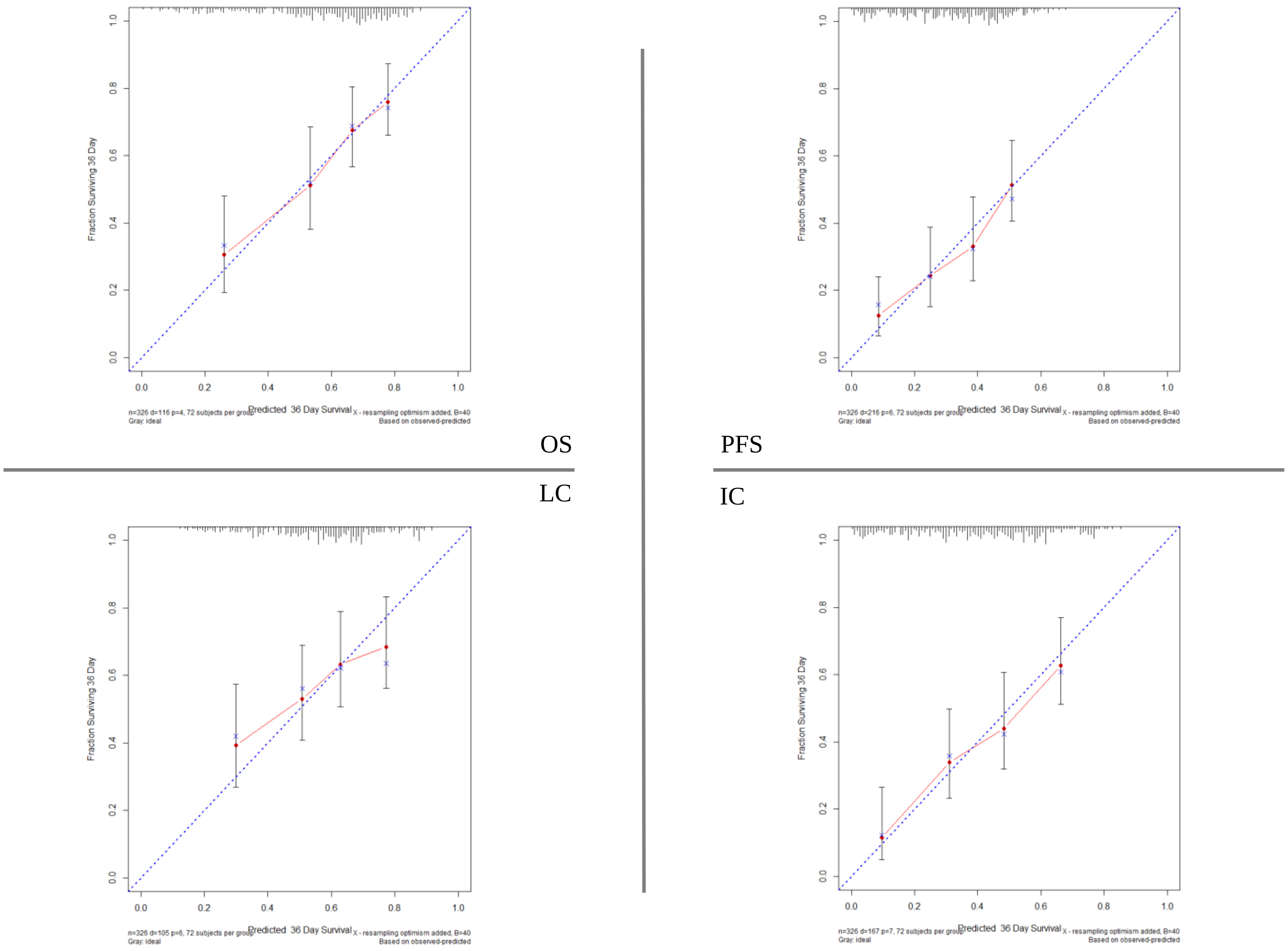

Fig S3: Histogram of propensity scores for patients between SBRT and TACE groups

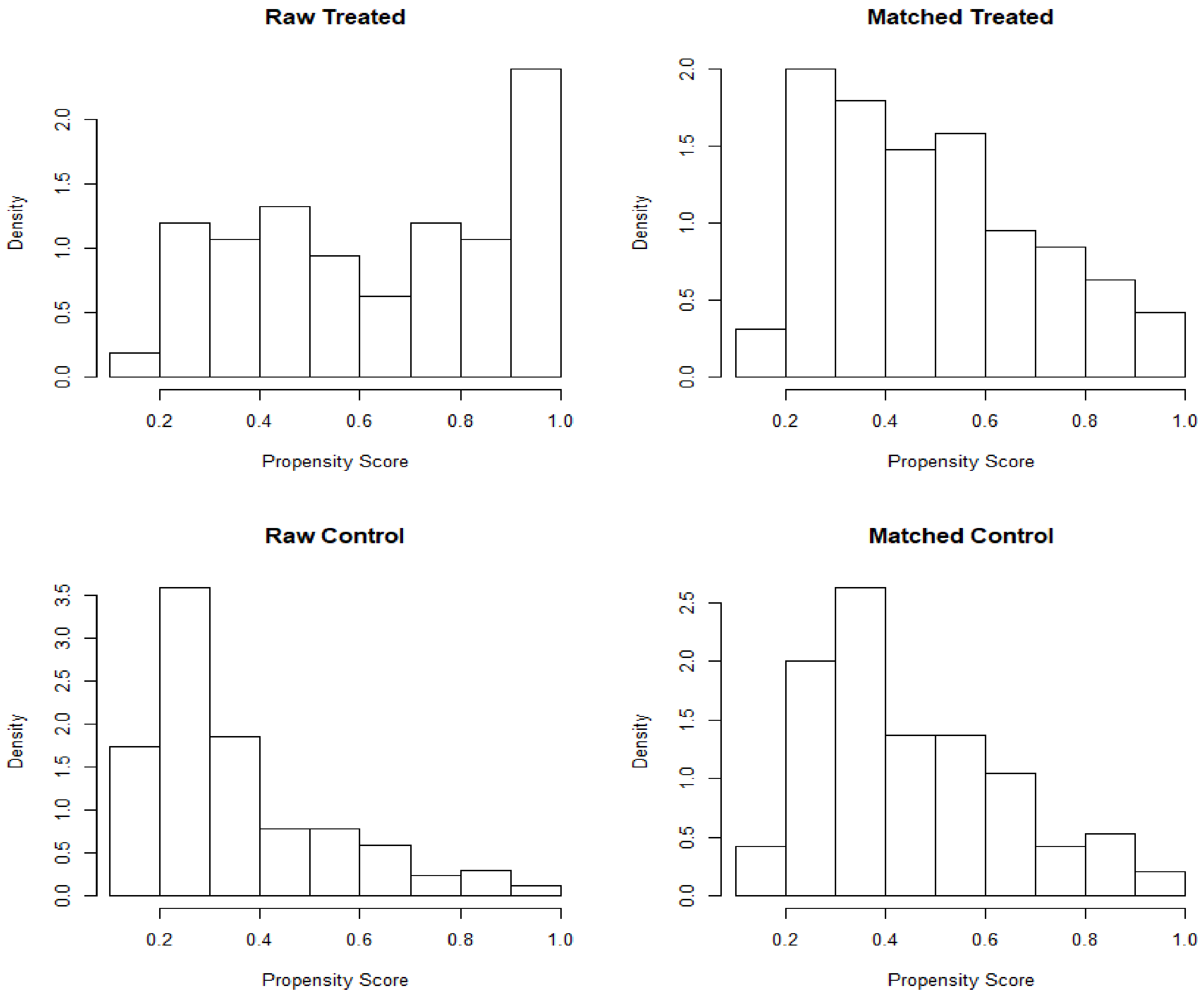

Supplement: Supplementary file 1 [file Data_Sheet_1.PDF]
